# Derivation of Thyroid Follicular Cells From Pluripotent Stem Cells: Insights From Development and Implications for Regenerative Medicine

**DOI:** 10.3389/fendo.2021.666565

**Published:** 2021-04-20

**Authors:** Alberto Posabella, Andrea B. Alber, Hendrik J. Undeutsch, Raoul A. Droeser, Anthony N. Hollenberg, Laertis Ikonomou, Darrell N. Kotton

**Affiliations:** ^1^Center for Regenerative Medicine, Boston University and Boston Medical Center, Boston, MA, United States; ^2^University Center of Gastrointestinal and Liver Diseases—Clarunis, University of Basel, Basel, Switzerland; ^3^Division of Endocrinology, Diabetes and Metabolism, Joan and Sanford I. Weill Department of Medicine, Weill Cornell Medicine, New York, NY, United States; ^4^The Pulmonary Center and Department of Medicine, Boston University School of Medicine, Boston, MA, United States; ^5^Department of Oral Biology, School of Dental Medicine, University at Buffalo, State University of New York, Buffalo, NY, United States

**Keywords:** thyroid follicular cells, congenital hypothyroidism, pluripotent stem cells, regenerative medicine, directed differentiation

## Abstract

Stem cell-based therapies to reconstitute *in vivo* organ function hold great promise for future clinical applications to a variety of diseases. Hypothyroidism resulting from congenital lack of functional thyrocytes, surgical tissue removal, or gland ablation, represents a particularly attractive endocrine disease target that may be conceivably cured by transplantation of long-lived functional thyroid progenitors or mature follicular epithelial cells, provided a source of autologous cells can be generated and a variety of technical and biological challenges can be surmounted. Here we review the emerging literature indicating that thyroid follicular epithelial cells can now be engineered *in vitro* from the pluripotent stem cells (PSCs) of mice, normal humans, or patients with congenital hypothyroidism. We review the *in vivo* embryonic development of the thyroid gland and explain how emerging discoveries in developmental biology have been utilized as a roadmap for driving PSCs, which resemble cells of the early embryo, into mature functional thyroid follicles *in vitro*. Finally, we discuss the bioengineering, biological, and clinical hurdles that now need to be addressed if the goals of life-long cure of hypothyroidism through cell- and/or gene-based therapies are to be attained.

## Introduction

The generation of thyroid cells *in vitro* from pluripotent stem cells (PSCs), such as embryonic stem cells (ESCs) or induced pluripotent stem cells (iPSCs), can provide access to an inexhaustible source of cells that has been previously difficult to stably maintain in culture if isolated from humans. Hence, these recently engineered cells hold promise for regenerative medicine applications. Primary thyroid tissue, especially human tissue, can be difficult to access in large quantities. In contrast, generating thyroid follicular progenitors from PSCs could potentially provide an unlimited supply of proliferating cells, which can also be genetically manipulated, employed for future *in vivo* transplantation, or used *in vitro* to study the molecular mechanisms underlying thyroid development and differentiation.

The gold standard stem cell-based model system used *in vitro* for many years to study embryologic development of a variety of lineages, including thyroid epithelial cells, are ESCs, pluripotent cells isolated and cultured from the inner cell mass of the blastocyst embryo. These cells are characterized by self-renewal capacity exemplified by their high proliferative potential and exhibit pluripotency, defined as the ability to differentiate into all tissues of the three embryonic germ layers: ectoderm, endoderm, and mesoderm (1–3).

Since ESCs are derived from the inner cell mass of the blastocyst embryo, their downstream progeny, if applied for clinical transplantation, would be allogeneic to the intended recipient, thus limiting their potential clinical applications based on immunogenicity (4–8). This hurdle motivated a decades long search for approaches to engineer PSCs from adult cell sources as successfully generating such cells might also facilitate transplantation of autologous PSC derivatives to reconstitute *in vivo* organ function. The advent of iPSC technology 15 years ago, represented a turning point in the history of stem cell biology, that allowed researchers to generate PSCs from post-natal somatic tissues and differentiate them into functional cell types (9). By transiently over-expressing four transcription factors (TFs), Oct4, Sox2, Klf4, and cMyc, Takahashi and Yamanaka reprogrammed somatic cells, such as fibroblasts, into the near-equivalent of ESCs, providing a stable, self-renewing source of PSCs, which they termed iPSCs, that could be made from mice (9) and later humans (10, 11) of any age.

In the years since their discovery, iPSCs have been generated by reprogramming easily accessible cells, including skin fibroblasts (9, 11) or peripheral blood, taken from patients with a variety of disease affecting nearly every organ system or lineage (12, 13), including patients with congenital hypothyroidism (14). While the generation and banking of normal or disease-specific iPSCs is no longer a hurdle (15–18) – a variety of iPSC bio-repositories having been established for research – it has become clear that the main hurdle to applying these cells for *in vitro* disease modeling or *in vivo* regenerative transplantation therapies is the limited capacity to effectively or reliably differentiate these PSCs into stable, functional, or mature downstream lineages.

Congenital and post-surgical hypothyroidism makes the thyroid follicular lineage a particularly relevant target for regenerative therapy. Generation of this lineage from PSCs, either unmodified or genetically corrected in the case of congenital hypothyroidism, by *in vitro* differentiation remains an attractive goal in order to accomplish future cell therapies. Moreover, the possibility to use disease-specific cell lines provide predictive models of the effects of inactivating or activating gene mutations in individual human beings, presenting unprecedented opportunities to understand mechanisms that control normal *vs.* aberrant early human thyroid development and permitting the advent of personalized therapeutic approaches to treat individual patients. Since PSCs represent a phenotype similar to the blastocyst embryo, an increasingly widespread approach to their successful differentiation *in vitro* has been recapitulating a sequence of embryonic developmental milestones *in vitro* in order to iteratively coax these cells to form sequentially more mature cell types (7). In order to successfully generate fully functional thyroid follicular cells, a deep understanding of the embryological sequence of development of the thyroid gland is thus critical. Unfortunately, this understanding of thyroid *in vivo* development has been lacking until recently; thus, severely limiting the pace of advances made in generating mature thyroid follicular cells from PSCs.

In the following sections, the key steps of embryonic thyroid development are reviewed, with an emphasis on their importance in designing differentiation approaches for generating functional mature thyroid follicular cells from PSCs *in vitro*. We review how the blastocyst embryo forms the primary germ layer, definitive endoderm, from which the thyroid gland primordium forms, the transcriptional circuitry and major signaling pathways that regulate this sequence, and how these genes and pathways have been stimulated in mouse or human PSCs *in vitro* in order to engineer thyroid cells *in vitro*. Finally, we review progress in generating reprogrammed iPSCs from mice or from patients with congenital hypothyroidism, their successful directed differentiation into definitive endoderm and downstream differentiated thyroid progeny, and recent progress in applying these derivatives for transplantation to reconstitute thyroid function *in vivo*.

## Transcriptional Regulation of Thyroid Development

### Thyroid Lineage Specification in the Ventral Foregut Endoderm

The thyroid hormone producing cells of the mature gland are thyroid follicular epithelial cells that originate from a small pool of progenitors in the foregut endodermal tube early in embryonic development. Other cells within the thyroid gland include C-cells, which produce calcitonin and are derived from a separate embryonic lineage. The origin of the first primordial thyroid follicular progenitors has been well documented through a variety of developmental studies using mouse, *Xenopus*, chick, and zebrafish embryos, because in all species, as in humans, the cells emerge from the definitive endoderm, a germ layer formed as the early blastocyst embryo undergoes gastrulation (14, 19–21). Thus, a review of methods used to differentiate stem cells into thyroid follicular cells must first begin with an explanation of the signals and genes that regulate gastrulation and how these same processes can be recapitulated *in vitro* to initiate the differentiation of PSCs in the laboratory.

Keller and colleagues first suggested using knowledge of *in vivo* embryonic development to replicate developmental processes *in vitro* through specific pathway activators or inhibitors, applied to cultured cells sequentially or combinatorially, at different dosages and exposure times (22). In a similar way, the *in vitro* differentiation of PSCs toward mature thyroid follicle cells can be “directed” by stepwise activation or inhibition of pathways and resulting expression of TFs that recapitulate normal thyroid development, beginning with gastrulation to differentiate blastocyst inner cell mass-like cells into primitive streak followed stepwise by definitive and foregut endoderm.

During gastrulation, region-specific activation or inhibition of different pathways such as bone morphogenetic protein (BMP), Wnt/β‐catenin and Nodal/transforming growth factor (TGF)-β promote the development of primitive streak from migrating cells of the epiblast, which originate from the inner cell mass of the blastocyst embryo. Subsequently the most anterior migrating cells within this primitive streak pattern, known as anterior primitive streak, in response to high levels of Nodal signals, upregulate the transcriptional program of definitive endoderm during the emergence of the three different germ layers: ectoderm, endoderm, and mesoderm (7, 23–29).

The development of the definitive endoderm, its folding into an endodermal gut tube, and its subsequent patterning into foregut, midgut and hindgut domains along an anterior-posterior (A-P) axis gives rise to an array of epithelial progenitors that contribute to the formation of the major endodermal organs such as thyroid, thymus, lungs, esophagus, stomach, liver, bile ducts, pancreas, and intestine. The thyroid represents the most anterior mid-line organ of these foregut endodermal derivatives (23, 26, 27).

Thus, a deep understanding of the roles of Wnt/β-catenin, BMP and Nodal/TGF-β signaling in the development of definitive endoderm from the primitive streak is critical for any successfully directed differentiation of PSCs.

A key inducer of definitive endoderm *in vivo* is the Nodal/TGF-β signaling pathway. A 2004 seminal paper first showed that high levels of Nodal/TGF-β signaling lead to induction of a gene regulatory network consisting of specific endodermal genes, such as *Foxa2*, while, low levels of Nodal/TGF-β signaling lead to a population of mesodermal cells characterized by the expression of markers such as Brachyury (*Bry*) (30).

In order to recapitulate these developmental stages *in vitro*, the same group used a double knock-in reporter mouse ESC line expressing CD4 linked to the *Foxa2* locus, and green fluorescent protein (GFP) targeted to the *Bry* locus. Using this approach, they found that Nodal/TGF-β signaling, induced by Activin A (hereafter Activin), is required for the generation of a Foxa2^CD4+^/Bry^GFP+^ population, which resembles the *in vivo* anterior primitive streak population. Subsequently, in response to a longer exposure to Activin, they were also able to induce a specific endodermal Foxa2^CD4+^/Bry^GFP-^ population, resembling definitive endoderm and indicating the successful recapitulation of gastrulation *in vitro* using ESCs (30, 31).

This discovery was a landmark for future studies focusing on endodermal downstream organs such as the thyroid, liver, pancreas or lung, allowing the directed differentiation of PSCs into definitive endoderm, using Nodal/TGF-β signaling activation by temporally defined exposure to Activin (29, 32). Moreover, a comparison of the relative equivalency of *in vitro* definitive endoderm derived from iPSC and ESC populations showed highly similar capacity to undergo directed differentiation into definitive endoderm progenitors (33).

In addition to determining the key inductive signals for the *in vitro* differentiation of PSCs towards definitive endoderm, an evaluation of the differentiation efficiency, and thus an estimate of how many cells are competent to undergo the subsequent differentiation steps, has been instrumental in optimizing most endodermal directed differentiation protocols, including those for thyroid differentiation. The most convenient method is flow-cytometry analysis based on surface markers, which can profile the cells at definitive endoderm stage without the need for knock-in reporters. Using gene profiling in the endodermal compartment Gouon-Evans et al. identified C-KIT/CXCR4 as such surface markers, and have shown that the use of antibodies specific for these proteins allows for monitoring the efficiency of the directed differentiation towards the endoderm (29).

Given that the thyroid gland is the most anterior midline organ that develops from foregut endoderm, the subsequent step in directed differentiation towards the thyroid lineage consists of promoting anterior foregut patterning. This is accomplished in part by inhibiting definitive endoderm posterior patterning, characterized by CDX2 expression, to instead favor anterior foregut endoderm (AFE) patterning, characterized by the expression of marker SOX2. In order to identify the key signals responsible for the differentiation from definitive endoderm to AFE, Green et al. tested various growth factors and pathway inhibitors and found that a combination of TGF-β inhibition and BMP inhibition seems to drive the differentiation of human PSCs into an AFE-like SOX2^+^/CDX2^-^ population (34). Thus, treatment of PSC-derived definitive endoderm *in vitro* with noggin or dorsomorphin and SB431542 to inhibit BMP and TGF-β signaling respectively, has resulted in an AFE-like cell population competent to become lung/thyroid progenitors characterized by the expression of genes common to both lineages (35).

In order to understand the pathways that subsequently drive AFE-like populations towards a thyroid fate, several studies hypothesized an active role for FGF and BMP signaling, originating in the adjacent mesoderm (36). Studies in mouse and zebrafish models suggested that BMP/FGF signaling, both instructive and permissive, drive a committed population in the ventral pharyngeal foregut endoderm to differentiate towards thyroid progenitor cells characterized by the expression of the homeodomain TF, *Nkx2–1* (also known as thyroid transcription factor-1; *Ttf1* or *Titf1*) and the paired box TF, *Pax8* (37, 38). The onset of co-expression of *Nkx2-1* and *Pax8* has been employed by several groups as the earliest marker of foregut cells undergoing lineage specification to a thyroid epithelial cell fate. In fact, the co-expression of these TFs appears to only occur in thyroid follicular cells. In 2015, our group employed these markers in *Xenopus* and mouse embryos *in vivo* and *ex vivo*, and in mouse and human PSCs *in vitro*, to reveal that combinatorial FGF and BMP signaling is necessary and sufficient to promote AFE lineage specification into thyroid cell fate (14). In these models these signals could be promoted by FGF2 and BMP4 ligands and the signaling cascades were dependent on PI3 kinase in the case of FGF signaling and on phosphorylation of SMADs 1/5/8 in the case of BMP signaling.

### Overview of Early and Late Thyroid Development

Essential loss-of-function studies that analyzed the impact of *Nkx2-1*^-/-^ and *Pax8*^-/-^ mutation in murine thyroid development, indisputably showed that *Nkx2-1*/*Pax8* gene functions are critical for thyroid organogenesis (39, 40). In particular, mutations causing altered or abrogated expression of one or both of these genes produce a variety of phenotypes, from alterations in thyroid morphogenesis (thyroid dysgenesis) to thyroid hypoplasia or absence of one thyroid lobe (hemiagenesis) (39, 41–43). In humans, *NKX2-1*, *PAX8*, and *FOXE1* mutations (another TF important for thyroid development, see below) also appear to be the cause of human congenital thyroid disease (44). *NKX2-1* mutations underlie Brain-Thyroid-Lung syndrome, a rare genetic disease, that is almost always characterized by congenital hypothyroidism (45, 46). Similarly, mutations resulting in reduced DNA-binding of *PAX8* have been found in patients with thyroid dysgenesis and resulting thyroid dysfunction (47) whereas *FOXE1* mutations are the genetic cause of the Bamforth-Lazarus syndrome, that has congenital hypothyroidism as one of its clinical manifestations (44, 48).

Thyroid progenitor cells are also characterized by the expression of two other TFs: the homeodomain TF, *Hhex* and forkhead domain TF, *Foxe1*. Even though each of these four TFs is expressed in several other embryonic tissues, their co-expression uniquely defines thyroid follicular epithelial progenitors (44, 49, 50). Although none of these TFs is necessary for thyroid specification, their expression is indispensable for progenitor proliferation, migration and subsequent differentiation to and maturation of thyroid follicular cells. At the specification stage, *Nkx2-1*, *Pax8*, and *Hhex* do not cross-regulate each other (19). At later stages, *in vivo* studies suggest reciprocal, hierarchical interactions between the four TFs as they directly or indirectly, regulate each other’s expression or autoregulate, as in the case of *Nkx2-1* and *Pax8*. Additionally, physical interactions between NKX2-1 and PAX8 may control expression of differentiation-related genes, such as *Tg*, in a synergistic manner (50). In contrast, *Foxe1* has a lower role in this hierarchy as its expression is initially dependent on *Pax8* (49).

**Figure 1:**
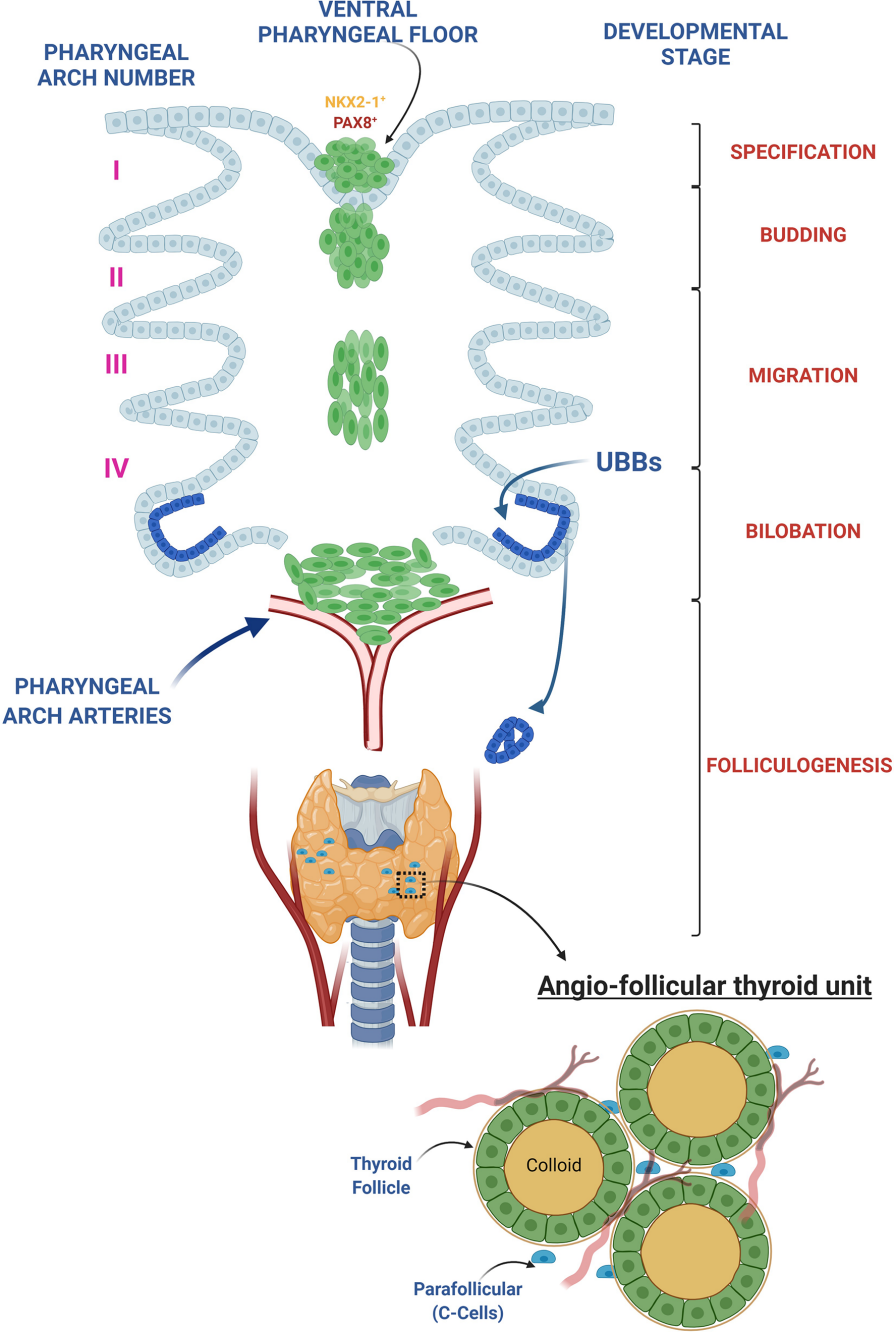
Thyroid development overview. The thyroid morphogenesis in mice can be divided in 5 different steps: *1*. Thyroid specification: occurs in the definitive endoderm-derived gut tube at embryonic day (E) 8.5 in mice and day post fertilization (DPF) 20-22 in human and is characterized by a thickening of a restricted area of the pharyngeal endoderm within the anterior ventral foregut epithelium, caudal to the first branchial branch, called the placode. The resulting foregut endoderm derived cells in the placode upregulate *Nkx2-1* and *Pax8*, the earliest known gene markers of “thyroid specification,” and represent the small pool of endodermal primordial thyroid progenitors from which the entire adult thyroid follicular epithelium will derive. *2*. Budding: a progressive increase in thickness of the placode determines an evagination of the pharyngeal endodermal tissue, firstly visible at day E9.5 in mice and DPF 24 in human. *3*. Migration: at E10-10.5 in mice and DPF 26 in human, the thyroid primordium begins its migration to pretracheal position. At the moment of detachment, the thyroglossal duct degenerates, leaving the thyroid bud completely surrounded by mesenchymal tissue, losing any contact with the floor of the pharynx. *4*. Bilobation: the earliest signs of the bilobation can be observed at E11.5 in mice, when the thyroid shape changes from spherical to oval but it is only at E12.5 in mice that the thyroid extends bilaterally along the third pharyngeal arch arteries reaching its definitive shape. In human the bilobation takes approximately 20 days, from DPF 28 until DPF 48. During this process the thyroid primordium fuses with paired ultimobranchial bodies (UBB), lateral anlagen originating from the fourth pharyngeal pouches, designated to a C-Cell (calcitonin-producing cell) fate. *5*. Folliculogenesis: once the bilobation is complete, the thyroid undergoes folliculogenesis. During this process, from E14.5 to E17.5 in mouse and from DPF 60 to DPF 84 in human, thyroid follicular epithelial cells (thyrocytes) acquire epithelial polarity and the follicular lumen gradually grows, resulting into the final shape of the follicle. Vascularized, polarized follicles comprise the functional and morphological thyroid unit, namely the angio-follicular thyroid unit.

As shown in **Figure 1**, during early development, the thyroid detaches from the ventral pharyngeal foregut endoderm and moves to the pre-tracheal position, where it reaches its definitive shape, consisting of two primitive lobes connected by a narrow isthmus. At this point, the thyroid undergoes a highly synchronized and coordinated process called folliculogenesis, where each progenitor cell undergoes cytoskeletal modifications at the apical and basolateral membrane, regulated by tight and adherens junction complexes, that guide epithelial cells to assemble together forming the definitive follicular shape (51–53).

**Figure 2:**
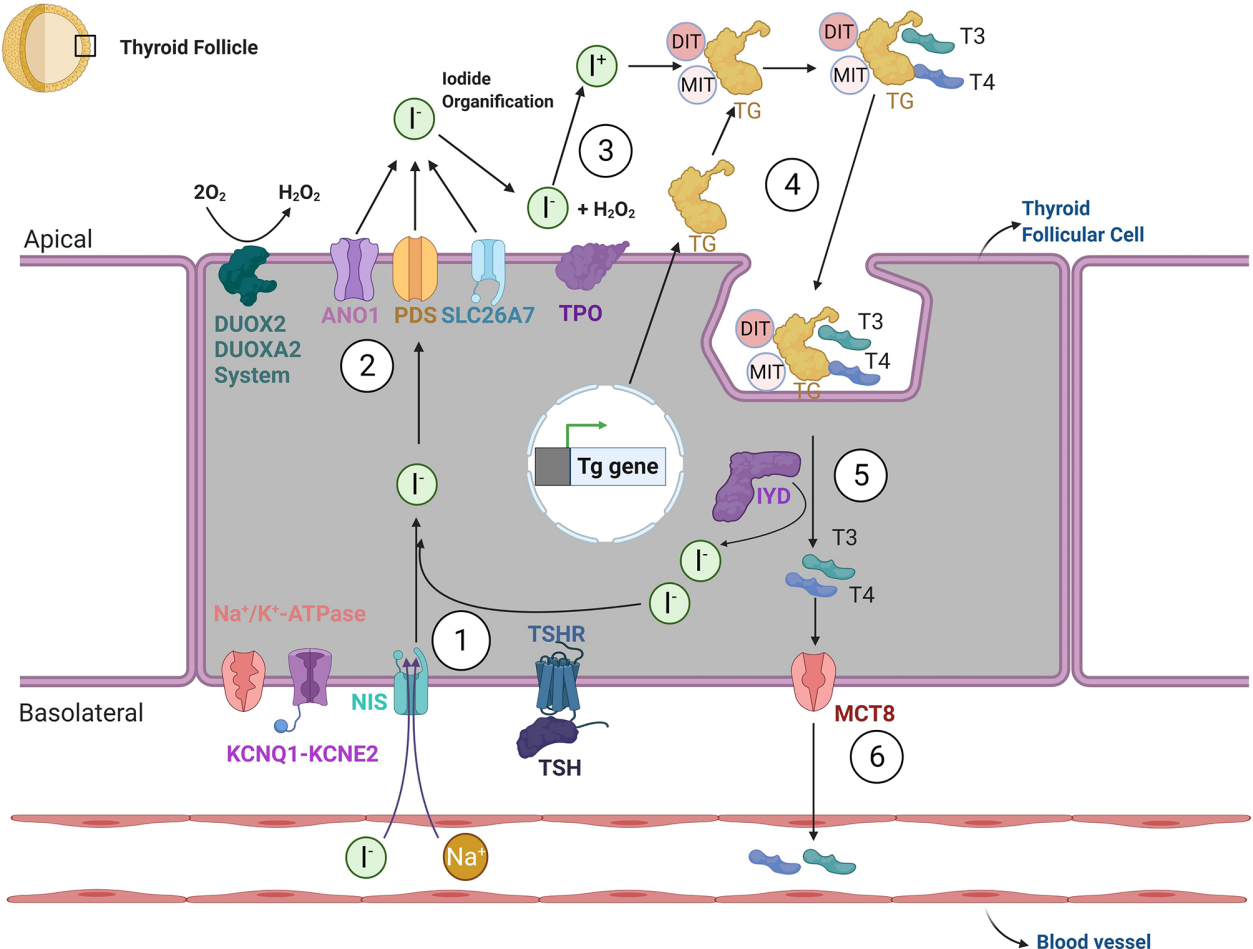
Thyroid hormone biosynthesis. The biosynthesis of thyroid hormones can be summarized through six steps. 1. Iodide reaches the cytosol of thyroid follicular cells *via* an active transport mechanism, mediated by sodium-channel iodine - Na+/I− - symporter (NIS; also known as SLC5A5). NIS activity is generated by the sodium gradient created by the Na/K-ATPase and KCNQ1-KCNE2 potassium channel. After crossing the basolateral membrane, iodide reaches the apical membrane after which it will undergo organification. The movement of iodine across the apical border is mediated by a passive transport system, through potentially the specific channels Pendrin (also known as PDS or SLC26A4) and ANO1 (Anoctamin-1). SLC26A7 may be an additional such transporter. 3. After crossing the apical membrane, the enzyme thyroid-peroxidase (TPO) oxidizes iodide to atomic iodine (I) using a source of hydrogen peroxide (H_2_O_2_) provided by dual function oxidases (DUOX2 and DUOXA2). 4. In the presence of iodide and H_2_O_2_, TPO catalyzes the iodination of thyroglobulin (TG) tyrosine residues, at the apical plasma membrane; both singly iodinated tyrosine residues (mono-iodotyrosine—MIT) and doubly iodinated tyrosine residues (di-iodotyrosine—DIT) are formed during iodination of thyroglobulin. 5. After the re-internalization of TG from the follicular lumen into the follicle, TG undergoes lysosomal degradation *via* proteolysis, which liberates thyroid hormones 3,5,3′-triiodothyronine (T3) and 3,5,3′,5′-tretraiodothyronine (T4). Surplus MITs and DITs are deiodinated by the iodotyrosine deiodinase 1 (DEHAL1/IYD) and iodine is transported to the intrathyroidal iodide pool 6. Finally, T4 and T3 exit the thyroid gland *via* the monocarboxylate transporter 8 (MCT8) and potentially other modalities. The transcription of specific thyroid genes and thyroid function *in vivo* is under the control of thyrotropin (TSH) *via* its protein G-coupled receptor (TSHR).

As a result of this process, the thyroid acquires its mature functional features characterized by: 1) active iodide uptake at the basolateral membrane of the follicular epithelium, mediated by Na^+^/I^−^ - symporter (NIS/SLC5A5); 2) synthesis of Thyroglobulin (TG) by follicular epithelial cells and 3) iodide oxidation regulated by thyroid-peroxidase (TPO) at the follicular apical membrane (54, 55). The chain of reactions responsible for the biosynthesis of the thyroid hormones triiodothyronine (T3) and thyroxine (T4) is summarized in **Figure 2**.

Lineage specification and proliferation of thyroid follicular epithelial cells from AFE precursors as well as initial follicle formation do not require thyroid-stimulating hormone (TSH) signaling, as shown by Di Lauro and colleagues using genetic mouse models (56), but subsequent follicular epithelial maturation and hormone production, including sufficient up-regulation of *Nis* and *Tpo* genes, depend on the activity of TSH. Therefore, requirements for TSH signaling appear to be quite distinct in the developing and adult thyroid gland (19, 56).

## Generation of Thyroid Cells From Pluripotent Stem Cells (PSCs)

### Use of Embryonic Stem Cells (ESCs) to Generate Thyroid-Like Cells

In a first attempt to induce thyroid follicular differentiation *in vitro*, Lin et al. generated embryoid bodies (EBs) from mouse ESCs in FBS-containing media and subsequently treated them with recombinant TSH. This led to some degree of expression of thyroid lineage markers such as *Pax8*, *Tshr, Tpo* and *Nis*, as detected by RT-PCR and immunofluorescence (57). Notably, levels of *Nkx2-1* expression were not measured. Given the absence of primary tissue controls (embryonic or adult thyroid tissue) and the undefined nature of the differentiation media the interpretation of these results is unclear.

In order to better quantify differentiation outcomes, Arufe et al. used a transgenic mouse ESC line expressing GFP under the control of the *Tshr* promoter, which allowed for the quantification of the temporal dynamics of *Tshr* promoter activation upon treatment with recombinant TSH (58). GFP expression peaked at day 4 of differentiation and subsequently decreased. GFP-positive cells were sorted and further differentiated with recombinant TSH on 2-dimensional (2D) Matrigel, a mouse tumor-derived analogue of basement membrane, for 21 days. Using this differentiation approach, the authors observed the formation of follicle-like clusters, with 1% of cells claimed to co-express NIS and GFP, and NIS localized to the plasma membrane. In addition, TSH-treated cultures expressed *Pax8*, *Tpo*, and *Tshr*. TSH-treated cultures appeared to display higher levels of iodide uptake than non-treated cultures. Of note, neither of these studies could detect expression of TG. Similarly to the previous study, the absence of primary thyroid tissue controls and *Nkx2-1* expression data make these results difficult to interpret.

Arufe et al. subsequently published a further refined protocol using the TSHR-GFP cell line (59). After 2 days of EB formation, they not only added TSH, but also Activin for 4 days to support endoderm differentiation. Cells were subsequently transferred to a medium containing TSH, Insulin and IGF-1 until day 16. This led to the expression of *Tshr*, *Foxa2* and *Sox17*, NIS and GFP as well as weak expression of TG. In contrast, *Tpo* was not expressed.

Importantly, while these studies attempted to derive thyrocyte-like cells from ESCs, they did not follow a directed differentiation protocol, and only one of them employed Activin, a key factor to induce definitive endoderm. The studies suffer from several limitations, such as the use of serum, inefficient induction of thyroid markers and the lack of in-depth characterization of differentiation outcomes by more quantitative methods such as RT-qPCR or genome-wide transcriptomics, by microarrays or RNA-sequencing. In addition, they mostly lacked comparison to primary controls. While the detection of certain thyroid markers suggested that *in vitro* differentiated cells had some thyroid-like features, the studies did not address their actual similarity to primary thyroid tissue, nor demonstrate whether they could synthesize thyroid hormones. Nevertheless, these early studies did show some initial promising results and thus served as important milestones for future studies.

### Forward Programming by Overexpression of Key Thyroid TFs

The early studies described above used recombinant proteins and growth factors such as TSH and Activin to induce PSCs to express some degree of thyroid epithelial-relevant genes *in vitro*, which, in the case of a successful differentiation protocol, would be associated with the expression of key thyroid TFs. An alternative approach is to directly induce the expression of those TFs by transient or constitutive overexpression. Forward programming of PSCs by TF overexpression has been successfully used for a variety of lineages (60–63). For the thyroid follicular lineage, this concept was first introduced by Antonica et al., who generated mouse ESC lines allowing for doxycycline-inducible forced overexpression of Nkx2-1, Pax8, or both Nkx2-1 and Pax8 (64). They first cultured those cells in hanging drops for 4 days to generate EBs, then transferred them to 3-dimensional (3D) Matrigel and added doxycycline for 3 days. Finally, they treated the cultures with recombinant TSH until day 22. Following transient, inducible expression of both Nkx2-1 and Pax8 from day 4 - 7 using this protocol, the authors observed a sustained upregulation of endogenous *Nkx2-1* and *Pax8* on day 22, as well as increased levels of *Tshr*, *Nis*, *Tpo* and *Tg* compared to untreated cells, as shown by qPCR. Cells formed follicular aggregates with NIS localizing to the basolateral membrane, TG localizing intracellularly and in the luminal compartment, and iodinated TG in the luminal compartment. Importantly, 60.5% of day 22 cells co-expressed NKX2-1 and PAX8, suggesting a much higher efficiency in thyroid follicular differentiation than had been achieved in previous studies. Of note, overexpression of Nkx2-1 alone led to similarly high levels of *Tshr*, *Nis*, *Tpo* and *Tg*, but cells failed to organize in 3D follicle-like structures. In contrast, overexpression of Pax8 alone could not induce expression of thyroid markers. The authors then aimed to assess the *in vivo* functionality of their *in vitro* derived follicular structures by grafting them under the kidney capsules of hypothyroid mice (induced by radioactive iodine). Follicles successfully integrated into the host niche and expressed NKX2-1, PAX8, FOXE1, cytosolic TG, basolateral NIS and luminal T4 four weeks after grafting. Most importantly, T4 levels in the plasma of recipients were restored, suggesting functional rescue of the hypothyroid phenotype.

Instead of a transient TF overexpression approach, Ma et al. used constitutive overexpression of Nkx2-1 and Pax8 (65). They generated mouse ESC lines stably overexpressing either Nkx2-1, Pax8, or both, by electroporation of ESCs with retroviral vectors. Overexpression of Nkx2-1 and Pax8 did not affect the expression levels of pluripotency markers. Single Nkx2-1 or Pax8 transfected cells showed a 5-10-fold induction of thyroid genes such as *Nis* (*Slc5a5*), *Tshr*, *Tg*, and *Tpo*, whereas double transfected showed a 30-55-fold increase. However, these levels were still substantially lower than expression levels in the thyroid cell line FRTL5. The authors then treated the cells with Activin for 5 days and subsequently cultured them on Matrigel coated dishes in the presence of TSH for 16 additional days. This led to a significant increase in *Pax8*, *Nkx2-1*, and *Foxe1* expression and robust activation of *Nis*, *Tshr*, *Tg*, and *Tpo* at similar levels as in FRTL5 cells, suggesting an activation of the thyroid transcriptional program. However, in contrast to the Antonica et al. study, the *in vivo* functionality of these cells has not yet been demonstrated.

### Recapitulating Thyroid Development by Directed Differentiation

While forward programming by TF forced overexpression has greatly increased the efficiency of thyroid differentiation and the quality of differentiation outcomes, its use for clinical applications is limited, as the use of transgenic cell lines is required. Thus, improving differentiation outcomes without the need for exogenous gene expression would be an important step towards successful clinical applicability of *in vitro* differentiation for regenerative medicine. One way to achieve this is by recapitulating developmental milestones when differentiating cells *in vitro* in defined culture conditions without the use of serum or exogenous transgenes. This has partially been done in the early studies mentioned above, for example by using factors such as Activin to induce endoderm differentiation.

One step towards this goal involves the stepwise use of serum-free media to coax PSCs through each directed differentiation developmental milestone on the path towards thyroid follicular cell differentiation. For example our group generated an Nkx2-1^GFP^ knock-in mouse ESC line, such that GFP is expressed from within the *Nkx2-1* genomic locus, in order to monitor differentiation towards lung and thyroid progenitors (35) by following a fluorochrome reporter. We first observed that when differentiating cells with Activin only, a substantial number of definitive endodermal cells co-express *Foxa2* and *Foxa3*, which suggests posterior patterning of the resulting endodermal gut tube derivatives and loss of competence to differentiate into anterior foregut endodermal lineages such as lung and thyroid, while retaining competence to form more posterior fates, such as liver. Based on a previous study focused on generating AFE from ESCs (34), we used Activin followed by exposure to BMP and TGF-β inhibitors (Noggin and SB431542 for 24 h; differentiation day 5 – 6). This minimized *Foxa3* expression, and thus patterned cells towards a more anterior foregut endodermal fate with competence to form Nkx2-1+ endodermal progeny in response to a medium containing a cocktail of candidate thyroid/lung inducing factors: FGF2, Wnt3a, FGF10, KGF, BMP4, EGF, and heparin (referred to as WFKBE+F2/H medium; until day 15). Using this protocol, 24% of cells were found to be GFP positive, as compared to less than 1% when using Activin without subsequent BMP/TGF-β inhibition. Importantly, *Nkx2-1* expression levels on day 15 were comparable to levels in E18.5 primary lung and thyroid tissue. In response to sustained FGF signaling, purified Nkx2-1^GFP+^ cells expressed a variety of lung epithelial markers such as *Sftpc*, *Cc10* (*Scgb1a1*) and *Foxj1*, as well as thyroid epithelial markers such as *Pax8*, *Tg* and *Tshr* implying the Nkx2-1+ endodermal progenitors generated in this complex protocol represented a heterogeneous mixture of thyroid and lung lineages. Exposure of the resulting cells to a “thyroid maturation media” containing TSH, NaI and IGF-1 led to an increase in *Tg* and *Tshr*, but did not change expression of lung markers. While the derivation, purification, and tracking of thyroid competent Nkx2-1+ endodermal progenitors engineered from ESCs represented an advance in directed differentiation approaches, at that time it could not be determined with the use of only single Nkx2-1^GFP^ reporter whether thyroid and lung lineages were emerging in separate progenitors, as would be expected in the foregut *in vivo*, or were artifactually co-expressed within the same ESC-derived cells.

To distinguish these possibilities, our group engineered a dual-fluorescence mouse PSC reporter system carrying reporters for *Nkx2-1* and *Pax8* loci in order to find that lung and thyroid Nkx2-1+ endodermal programs were expressed in separate ESC-derived foregut endodermal lineages *in vitro*. This result then provided a rationale for seeking to identify lineage specifying factors that would promote the separate emergence of each lineage in an effort to engineer pure thyroid follicular progenitors without the presence of lung progenitors (14). In order to identify the essential factors for thyroid differentiation we performed a sequential withdrawal of individual factors from our previously published WFKBE+2 cocktail and found that BMP4 and FGF2 were indispensable for induction of *Nkx2-1/Pax8* co-expression. A medium supplemented with only BMP4 and FGF2 promoted the emergence of thyroid progenitors from PSC-derived AFE, which could be sorted to purity and exhibited competence to upregulate the thyroid differentiation markers *Tg*, *Tshr*, and the maturation markers *Slc5a5* and *Tpo*. To assess whether these findings gleaned from the mouse ESC model system reflected the same lineage specifying pathways that regulate thyroid development *in vivo* we examined developing *Xenopus* and mouse embryos and cultured embryonic foregut explants from each species ex vivo. We found that incubation of foregut explants from E8.0 mouse embryos, as well as *Xenopus* embryos with either BMP inhibitors or FGF inhibitors blocked induction of *Nkx2-1* and *Pax8* in the region of the thyroid primordium and abrogated expression of thyroid TFs. In contrast, inhibition of Wnt, retinoic acid and VEGF signaling had no effect. In contrast, removal of Wnt blocked lung lineage specification while leaving thyroid specification intact, thus establishing that combinatorial BMP and FGF signaling promotes AFE specification into thyroid whereas combinatorial BMP and Wnt signaling promotes AFE specification into lung.

Once thyroid lineage specifying factors had been identified, we were able to further optimize the directed differentiation protocol using TSH and culture in 3D Matrigel to promote thyroid maturation and organoid formation. In this optimized protocol cells were specified from mouse ESC-derived AFE in FGF2 and BMP4 from day 6 – 12. Nkx2-1 positive cells were then sorted and further differentiated in serum-free media in 3D conditions supplemented with a variety of growth factors including TSH (FGF2, FGF10, EGF, IGF-1, insulin, and TSH until day 20 and FGF2, FGF10, EGF, IGF-1, insulin, transferrin, selenium, and TSH until day 26). A variety of conditions that contributed to maturation were evaluated in these studies, including corticosteroids (dexamethasone), TSH dosing, and promoters of cyclic AMP signaling (14). By the end of the month-long protocol, 3D organoids were produced from mouse PSCs in entirely serum-free conditions and exhibited molecular features typical of thyroid follicles, including monolayered epithelia co-expressing NKX2-1 and PAX8 and luminal TG. Organoids were able to produce small amounts of T4 hormone *in vitro* consistent with their ability to organify iodide. Most importantly, we tested the *in vivo* functionality of these ESC-derived follicular organoids by transplanting them underneath the kidney capsules of radioactive-iodine-treated hypothyroid mice. T3 and T4 levels of recipient mice returned to normal levels within 4 weeks and importantly re-established the set point of the hypothalamic-pituitary-thyroid axis consistent with their ability to respond to TSH *in vivo*. Furthermore, as expected the transplanted thyroid organoids could concentrate iodide. Taken together these data demonstrated that *in vitro* generated organoids exhibit *in vivo* functional potential and are able to rescue hypothyroid mice.

### Human Directed Differentiation of PSCs to Produce Normal or Patient-Specific Thyroid Follicular Progenitors

For mouse directed differentiation protocols to be relevant for human therapies, discoveries made in murine, *Xenopus*, or other model systems need to be adapted for engineering human thyroid lineages. Importantly the culture conditions established by Kurmann et al. have been readily adapted to accomplish the serum-free directed differentiation of human ESCs and iPSCs to similarly generate NKX2-1+/PAX8+ thyroid follicular epithelial progenitors. The same lineage specifying factors used to differentiate mouse PSCs into endoderm, foregut endoderm, and then thyroid progenitors appear to be conserved in human development, with combinatorial BMP and FGF signaling also appearing to promote human thyroid lineage specification from AFE precursors (14, 66). Despite the very different gestational periods for mice *vs.* humans and although the dosing of growth factors and inhibitors in the human system differs slightly from mouse, similar timing of cell fate decisions can be induced in PSCs from each species *in vitro*, demonstrating evolutionary conservation of mechanisms regulating thyroid development, and producing human thyroid progenitors from human PSCs within a 2-week time frame, similar to differentiating mouse PSCs (14, 66, 67). After culturing these human PSC-derived thyroid progenitors further in the same maturation media used for mouse cells in Kurmann et al., we observed increased expression of early key thyroid markers such as *NKX2-1*, *PAX8*, *FOXE1*, *HHEX* and late markers such as *TG*, *TPO*, *TSHR*, *NIS* (*SLC5A5*) and *TSHR*. However, expression was still lower than in primary human adult thyroid tissue.

Fluorochrome reporters targeted to the human NKX2-1 locus in human iPSCs by gene editing have also been employed in proof of concept experiments to track, sort, and purify iPSC-derived human thyroid progenitors (67). To ensure these approaches are relevant to thyroid disease-specific cells, iPSCs made by reprogramming cells taken from multiple patients with the Brain-Thyroid-Lung syndrome and congenital hypothyroidism resulting from *NKX2-1* heterozygous mutations have been differentiated *in vitro* into thyroid progenitors using the protocols discovered in the above mouse ESC models (14). Similar to their mouse ESC study, Ma et al. have also generated human ESC lines stably overexpressing NKX2-1 and/or PAX8 and have used Activin and recombinant TSH to differentiate them towards a thyroid fate (68). In line with their findings in mice, they observed expression of thyroid-specific genes and formation of follicle-like structures with luminal TG expression, but lack of comparison to adult human thyroid controls have limited the interpretation of these expression levels.

Although a growing number of publications from us and others report the derivation of cells expressing some thyroid markers from human ESCs or iPSCs *in vitro*, to date the same level of functionality or maturation established for mouse derivatives has yet to be demonstrated for these human cells. Future work will likely focus on further optimizing approaches for the purification and maturation of human iPSC-derived thyroid cells. Transplantation to demonstrate *in vivo* function of human iPSC-derived follicular cells remains a high and unresolved hurdle on the path to developing thyroid regenerative therapies. The potential for PSCs to form teratomas *in vivo* if transplanted before full endodermal differentiation has been detected in some studies (33) and thus remains an important concern that will need to be extensively tested once functioning human thyroid lineages have been produced for *in vivo* testing in animal models. Although neither Antonica et al. nor Kurmann et al. observed any teratomas arising *in vivo* after transplantation of mouse ESC-derived thyroid follicular cells, extensive follow up times after transplantation still need to be examined to fully screen for this potential outcome.

### Combining Directed Differentiation With TF Overexpression

In order to study the mechanistic interplay between transient TF overexpression and developmental stages in directed thyroid differentiation, Dame et al. combined the differentiation protocol previously established by Kurmann et al. with pulsed overexpression of Nkx2-1 at different steps of the protocol (69). In this work, we used a mouse ESC line with a doxycycline-inducible Nkx2-1 transgene. These cells were differentiated towards the thyroid lineage by LIF withdrawal from day 0 - 2, Activin from day 2 - 5, Noggin and SB431542 from day 5 - 6, and FGF2 and BMP4 from day 6 - 14. Without Nkx2-1 induction this protocol yielded 7.5 – 10% Nkx2-1 positive cells. We then added 24 h pulses of doxycycline at different time-points: day 0 - 1, day 5 - 6, day 6 - 7, and day 7 - 8. While transgene activation by doxycycline resulted in an increase of NKX2-1-positive cells to 90 - 95%, these levels rapidly decreased upon removal of doxycycline, except for the day 6 - 7 AFE group, where sustained high levels of endogenous *Nkx2-1* were observed, and about 75% of cells remained NKX2-1-positive on day 14. When cultured in 2D in presence of FGF2 and FGF10 from day 14 – 22, cells treated with doxycycline from day 6 -7 expressed significantly higher levels of early thyroid markers (*Nkx2-1*, *Pax8*, *Foxe1*, *Hhex*, *Iyd*) and late thyroid markers (*Tg*, *Tpo*, *Nis*, *Tshr*) than non-induced cells. Importantly, the expression levels of most markers closely resembled levels in E13.5 Nkx2-1+ thyroid and adult thyroid primary tissues. When cultured in 3D Matrigel according to the Kurmann et al. protocol until day 30, cells formed follicular structures with typical thyroid molecular signatures, including basolateral NIS and luminal TG expression. When cultured up to day 50, cells exposed to iodide produced T4 hormone, at levels similar to native mouse thyroid tissue. Importantly, omission of anteriorization at the definitive endoderm stage led to a dramatic decrease in the number of NKX2-1-positive cells, and withdrawal of FGF2 or BMP4 resulted in reduced expression of *Nkx2-1*, *Pax8* and *Foxe1*. Thus, appropriately patterned AFE in combination with essential signaling pathways could undergo efficient and robust thyroid specification in response to transient Nkx2-1 induction, implying that an appropriate developmental epigenetic state is likely required for optimal thyroid competence of PSCs, even when engineered by TF over-expression. The derivation of thyrocytes *via* forward programming of mouse ESCs by longer (3-day) Nkx2-1/Pax8 overexpression (64) may lead to a similar functional differentiation outcome but it neither delves into mechanistic aspects of thyroid competence and cell fate decisions nor uses a developmentally relevant approach.

## Gene Expression Profiling of *in Vitro* Differentiated and Primary Thyroid Lineage Cells

Most studies discussed above have used traditional methods such as RT-qPCR and immunofluorescence to quantify the *in vitro* expression of certain selected thyroid-specific genes in differentiated cell populations and thus evaluate differentiation outcomes. In vitro differentiated cells can be characterized on a more global level by genome-wide gene expression profiling using methods such as microarrays, population-based (bulk) RNA-sequencing, or single cell RNA-sequencing. Apart from providing a much broader gene expression profile than low- to medium-throughput methods such as RT-qPCR, genome-wide methods such as RNA-sequencing can also be applied to directly compare gene expression profiles of *in vitro* differentiated cell populations to *in vivo* references, for example by using methods such as linear algebra-based projections (70, 71). Furthermore, global gene expression profiling can identify novel signaling pathways or surface markers specific to a lineage of interest, which might help to further improve and refine *in vitro* differentiation protocols, as well as enable easier isolation of cell populations of interest.

Whereas a variety of gene expression profiling datasets already exist for other endodermal lineages such as the lung (72, 73), these methods have only recently begun to be applied to the thyroid lineage. The first study where gene expression profiling was used to compare primary lung and thyroid progenitor cells was published in 2011 (74). The authors used microdissection to isolate E10.5 lung and thyroid progenitor cells and analyzed them using microarrays. The authors identified transcripts specifically expressed in lung *vs.* thyroid buds, as well as enriched transcripts common to both lineages. In addition, they described *Bcl2* as an important anti-apoptotic factor that ensures cell survival specifically in the thyroid.

The first global gene expression profiling of *in vitro* differentiated thyroid lineage cells derived from PSCs was published by Dame et al. (69). In this study, we performed RNA-sequencing on 3 cell populations at different days of our thyroid directed differentiation protocol (see previous section for timing of transgene Nkx2-1 induction): doxycycline-induced day 1 cells (exit from pluripotency, minimal thyroid competence), doxycycline-induced day 7 cells (AFE stage, maximum thyroid competence) and day 14 cells, doxycycline-induced day 6 – 7 (NKX2-1-induced thyroid progenitors). The sequencing data confirmed the importance of active BMP and FGF signaling pathways on day 7 and day 14. Gene ontology showed a strong segregation of processes relating to differentiating cells and comparison with known cell types based on gene expression (using linear algebra-based projections) revealed that day 14 cells closely resemble purified E13.5 mouse Nkx2-1^GFP+^ thyrocytes, whereas day 1 cells projected strongly on mouse ESCs. In addition, we identified potential cell surface markers at different developmental stages, such as EPHB2 and SIRPA which were highly expressed at the definitive endoderm stage, and *Col25a1*, which was enriched in both induced and purified *in vitro* Nkx2-1+ thyroid progenitor populations.

A further important dataset was generated by Serra et al., who treated *in vitro* mouse ESC-derived AFE (day 6) cells with BMP4 and FGF2 to stimulate the specification of Nkx2-1-positive thyroid progenitors *vs.* BMP4 and Wnt3a to stimulate the specification of day 14 Nkx2-1-positive lung progenitors (66). Microarray analysis identified genes that were differentially expressed in the Nkx2-1-positive lung *vs.* thyroid progenitor populations. As expected, the thyroid progenitors were enriched for the key thyroid TFs *Pax8*, *Foxe1* and *Hhex*, as well as for the thyroid marker *Tg*. In addition, the thyroid progenitors were enriched for transcripts previously shown to be enriched in adult thyroid epithelial cells, such as *Prlr* and *Cd36*, as well as genes known to play a role in thyroid hormone biogenesis, such as *Iyd*, *Slc16a2* and *Duoxa2*.

A more recent gene expression dataset of interest (75), focused on *in vivo* Nkx2-1+ developing progenitor populations of the fetal lung (E9.0), thyroid (E13.5) and brain (E9.0). Cells from each tissue were isolated from mouse embryos at defined developmental stages, and their expression profiles were compared to each other and to their precursor populations (foregut endoderm and embryonic ectoderm). Analysis of developing thyroid epithelial cells confirmed enriched expression of *Pax8* and *Hhex*, as well as the early thyroid epithelial marker *Prlr*. Importantly, the study also confirmed *in vivo* expression of the putative thyroid markers *Aqp1* and *Col25a1*, which were previously shown to be enriched in mouse PSC-derived thyrocytes *in vitro* (69). Furthermore, FGF-dependent PI3K signaling was found to be specific to thyroid progenitors, confirming previous findings by Kurmann et al. (14).

**Figure 3:**
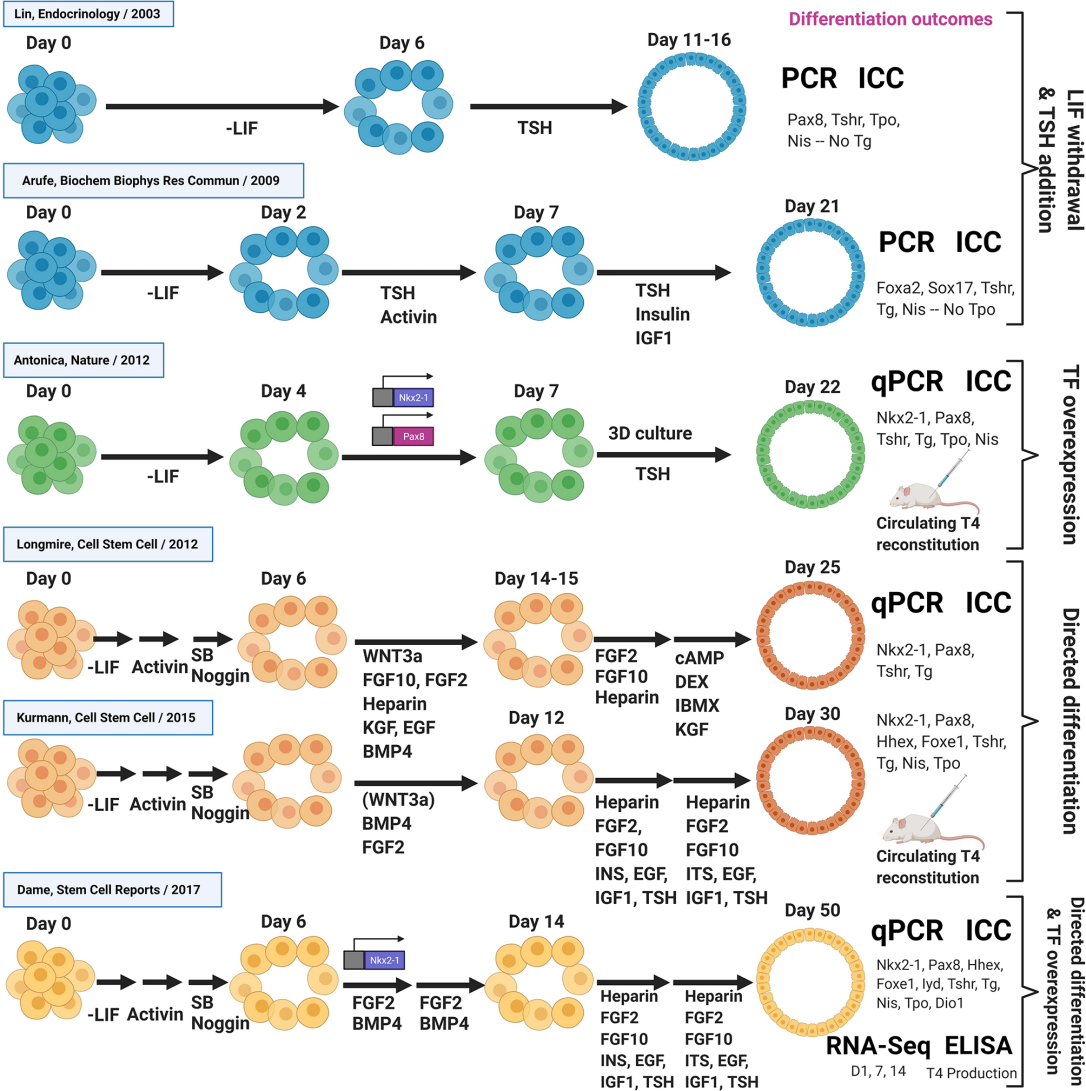
Overview of thyroid differentiation protocols. Overview of published *in vitro* differentiation approaches to generate thyroid lineage cells from PSCs. Schematic shows key signaling molecules and growth factors, as well as the methods used to quantify differentiation outcomes. In vitro differentiation approaches are sub-divided into early studies involving LIF withdrawal/TSH addition, TF overexpression, directed differentiation approaches, as well as studies combining directed differentiation with TF overexpression. All studies were performed with mouse PSCs, except from the Kurmann et al. paper that also included work with human PSCs. DEX, Dexamethasone; IBMX, 3-isobutyl-1-methylxanthine, a non-specific inhibitor of cAMP and cGMP phosphodiesterases; SB, SB-431542, a chemical inhibitor of the activin receptor-like kinase receptors, ALK5, ALK4 and ALK7 (TGF-β signaling inhibitor); PCR, RT-PCR; qPCR, RT-qPCR; ICC, Immunocytochemistry.

An overview of published *in vitro* differentiation approaches to generate thyroid lineage cells from PSCs, described so far, is summarized in **Figure 3**.

## Thyroid Gland Generation by Blastocyst Complementation

A novel approach for the generation of functional tissues, called blastocyst complementation, has been recently reported. This technique involves the intra- or interspecies injection of donor PSCs into blastocyst-stage recipient embryos resulting in the development of specific tissues/organs. The procedure requires animals lacking a particular tissue/organ as a result of a genetic deletion of genes indispensable for that lineage formation. The blastocyst obtained from these mutant animals, is injected with PSCs from a donor, not necessarily of the same species, that contains normal copies of these essential genes. Thus, PSCs-derived from the donor can have high contribution (chimerism) to form the missing tissue/organ in the recipient embryo.

Since Kobayashi et al. firstly generated rat PSC-derived pancreas cells in mouse embryos that carried gene deletions of specific TFs critical for pancreas development (76), a growing number of investigators have used this approach to efficiently generate different tissues, such as brain structures, kidneys, lungs, and blood vessels (77–80).

Recently, Wen et al. used intra-species blastocyst complementation to produce ESC-derived lungs and thyroids in *Nkx2-1*^-/-^ mouse embryos lacking these tissues; interestingly, histological analysis of these embryos at E17.5 confirmed the efficient restoration of thyrocyte progenitor cells, expressing both NKX2-1/PAX8 (81). In a similar vein, Ran et al. showed high thyroid contribution of donor ESCs and restoration of thyroid gland size and function in *Fgf10* Ex1^mut^/Ex3^mut^ mice that lack *Fgf10* expression (82).

According to these promising results, this approach could be used in the future to generate xenogeneic (human) thyroid glands in animal recipients, opening new horizons related to organ-replacement therapies.

In conclusion, the study by Wen et al. provides a proof-of-concept that thyroid epithelial cells can be efficiently restored by blastocyst complementation but, further ethical and scientific questions, such as functionality of the chimeric organ, efficiency of donor cell engraftment, and the relevance of any contributions of human cells to animal cerebral tissues will need be addressed before the feasibility and safety of blastocyst complementation as a strategy for thyroid regenerative medicine can be further advanced (83).

## Future Clinical Applications: Disease Targets and Technical Challenges

Congenital hypothyroidism is the most common congenital endocrine disorder and results in insufficient thyroid hormone secretion detected at birth or postnatally in approximately 1/3500 births. Although a small percentage of the causes of congenital hypothyroidism have been clearly ascertained, in the majority of the cases, congenital hypothyroidism is linked to a pathological ectopic thyroid gland location, frequently in a sublingual position without any gene mutation detected (84–88).

Despite the introduction of neonatal screening and the availability of thyroid hormone replacement therapy, consistent data have clearly shown that these children develop cognitive and motor function disabilities and social and emotional disorders requiring lifelong engagement of pediatricians and endocrinologists (89–91).

In adults, specific thyroid cancers and symptomatic bilateral goiter are responsible for permanent hypothyroidism, as a result of curative total thyroidectomy. Lifelong exogenous administration of thyroid hormones is the only curative treatment for those patients (92). Despite hormone replacement therapy, clear evidence has shown that many of these patients experience typical hypothyroid symptoms such as lethargy, depression, memory problems, cold intolerance and body weight gain (93, 94). Whether carcinogenesis recapitulates developmental processes is a frequent question in many cancers that arise from various endoderm-derived epithelia (95). Yet it is still not clear whether this is the case for thyroid carcinomas, as genetic instability and mutations are frequently drivers of proliferation rather than activators of recapitulated development. The presence of oncogenic genetic changes in thyroid cancers also prevents the use of resected thyroid tissue from these individuals from being employed as a transplanted cell therapy to reconstitute thyroid function

Considering these unsolved issues, a patient-tailored source of iPSCs obtained by reprogramming easily accessible cells, such as peripheral blood, followed by *in vitro* differentiation into thyroid cells and autologous transplantation represent potentially attractive therapeutic alternatives and a potential cure for hypothyroidism after a single treatment; however, there are still many technical challenges to overcome before these cells can be used for regenerative medicine.

Once robust, reproducible, and high-efficiency protocols for the derivation of functional thyrocytes from human PSCs are in place, a series of engineering challenges will likely arise as the field moves from the laboratory to the clinic. Fully-automated bioreactor systems, traditionally employed by the biotechnology industry for production of biopharmaceuticals, have been adopted for the derivation, expansion and differentiation of human PSCs (96). Such systems should be easily adaptable to the production of human iPSC-derived thyrocytes under current Good Manufacturing Practices (cGMPs) in clinically meaningful numbers (in the order of hundreds of millions).

The effect of organization of primary thyrocytes in 3D follicle-like structures on establishment of polarity and enhanced functionality, including iodide uptake, organification and hormone secretion, has been long recognized (97–100). This effect has been recapitulated in PSC directed differentiation protocols by the use of Matrigel for the 3D organization and maturation of thyroid progenitors (14, 64, 69). Yet, the clinical translation of such protocols will most certainly require the use of defined matrices of non-animal origin and this will be a future research area at the interface of biology and biomaterials science. Additionally, proof-of-principle studies using technologies such as decellularized native thyroid scaffolds (101), 3D bioprinting (102), and cell sheet engineering (103) have demonstrated the possibility of producing complex, organ-like thyroid constructs for future transplantation. Since the thyroid gland is an endocrine organ, transplanted thyrocytes do not necessarily require orthotopic transplantation to perform their function. Cell encapsulation using various biocompatible materials (alginate, agarose, and cellulose) has been studied for the delivery of other endocrine cell types, such as pancreatic islet β-cells (104). It can then be readily used for delivery of iPSC-derived thyroid follicles to blood-vessel-rich sites, enabling immunoisolation of the transplanted cells and unencumbered nutrient exchange and hormone secretion.

## Conclusions

The development of regenerative therapies based on PSC technology is quickly becoming a reality due to rapid advances being made in laboratory and developmental biology research. Indeed, the first clinical transplantation tests have already launched to evaluate the safety and potential regenerative capacity of iPSC-derived cells, for example in patients suffering from macular degeneration or Parkinson’s Disease (105, 106). A growing number of protocols are now available for the derivation of *in vitro* thyroid follicular epithelial cells from mouse and human PSCs; yet, there are still several hurdles to overcome before these cells can be used for clinical thyroid regenerative applications.

Two major current limitations involve the low or uncertain efficiency of PSC *in vitro* differentiation into thyroid cells, as well as the need for transgenic cell lines. Exogenous TF overexpression has so far yielded the most efficient thyroid progenitor specification protocols, and has also been very useful to understand how cell competence changes at each developmental stage. However, such transgenic cells have limited use for clinical applications. While we have shown that it is possible to induce a thyroid developmental program solely by using growth factor-supplemented media, this approach is still inefficient and relies on the use of genetically modified (knock-in) PSC reporter lines to isolate thyroid cells based on fluorescent markers. Therefore, one important step towards clinical application will be to find surrogate cell surface markers by which thyroid progenitors can be identified and purified without the need for knock-in reporter constructs. As already discussed, genome-wide sequencing approaches will be very useful to screen for these potential markers as well to compare the engineered thyroid cells to primary control cells head-to-head.

Another important milestone will be to successfully generate functional thyroid follicular epithelial cells from human PSCs. So far, the vast majority of studies focusing on *in vitro* differentiation of PSCs towards the thyroid lineage have used mouse ESCs. In contrast, only a few have focused on the human system and no study to date has profiled the functional or transplantable capacity of the putative human thyroid derivatives. Thus, while this initial data is encouraging, careful characterization will be required to establish a successful directed differentiation protocol for human thyroid progenitors.

In summary, the quickening pace of stem cell research is yielding an increasing number of exciting reports suggesting that stem cell-based therapies designed to regenerate *in vivo* thyroid function may be within reach in the years ahead. While it is difficult to estimate the time-frame needed to surmount the remaining scientific hurdles identified in this review before clinical trials can be safely attempted, some form of PSC-derived thyroid follicular epithelial cell preparation is likely to culminate in a transplantable preparation whose future thyroid reconstituting capacity can be tested *in vivo*, perhaps in the context of treating congenital or post-surgical hypothyroidism. The historical lessons summarized in this review suggest that a substantial investment in thyroid developmental biology research employing a variety of basic model systems from *Xenopus* to mice to humans is likely to help further propel the pace of discoveries that should lead to these future human clinical applications.

## Author Contributions

AP, ABA, LI, and DNK: contributed to the design, writing, and editing the manuscript and the figures. HJU, RAD, and ANH: contributed with critical revision of the manuscript. All authors contributed to the article and approved the submitted version.

## Funding

The authors are supported by the following grants: DNK by grants U01TR001810, R01DK105029, and N01:75N92020C00005; ANH and HJU by grant R01DK105029; LI by grant R01HL124280 and University at Buffalo (UB) Research Foundation Start-up Funds; AP by Swiss National Science Foundation (SNSF) P400PM_191038/1, Universität Basel Förderung des akademischen Nachwuchsförderung 3MS1042, Personenförderung Department of Surgery - Universität Basel, G&J Bangerter-Rhyner Stiftung, and Freiwillige Akademischen Gesellschaft (FAG); and ABA by Swiss National Science Foundation (SNSF) P2ELP3_191217.

## Conflict of Interest

The authors declare that the research was conducted in the absence of any commercial or financial relationships that could be construed as a potential conflict of interest.
